# Using phosphoproteomics data to understand cellular signaling: a comprehensive guide to bioinformatics resources

**DOI:** 10.1186/s12014-020-09290-x

**Published:** 2020-07-11

**Authors:** Sara R. Savage, Bing Zhang

**Affiliations:** 1 Department of Biomedical Informatics, Vanderbilt University, Nashville, TN USA; 2 Lester and Sue Smith Breast Center, Baylor College of Medicine, Houston, TX USA; 3 Department of Molecular and Human Genetics, Baylor College of Medicine, Houston, TX USA

**Keywords:** Phosphoproteomics, Bioinformatics tools, Kinase, Phosphorylation, Signaling

## Abstract

Mass spectrometry-based phosphoproteomics is becoming an essential methodology for the study of global cellular signaling. Numerous bioinformatics resources are available to facilitate the translation of phosphopeptide identification and quantification results into novel biological and clinical insights, a critical step in phosphoproteomics data analysis. These resources include knowledge bases of kinases and phosphatases, phosphorylation sites, kinase inhibitors, and sequence variants affecting kinase function, and bioinformatics tools that can predict phosphorylation sites in addition to the kinase that phosphorylates them, infer kinase activity, and predict the effect of mutations on kinase signaling. However, these resources exist in silos and it is challenging to select among multiple resources with similar functions. Therefore, we put together a comprehensive collection of resources related to phosphoproteomics data interpretation, compared the use of tools with similar functions, and assessed the usability from the standpoint of typical biologists or clinicians. Overall, tools could be improved by standardization of enzyme names, flexibility of data input and output format, consistent maintenance, and detailed manuals.

## Background

Kinase signaling, the reversible enzymatic addition of a phosphate group to a substrate, is an essential part of cellular activity. Because its dysregulation contributes to many diseases, numerous clinical trials have been performed with kinase inhibitors resulting in over 50 FDA-approved small molecules and targeted antibodies [1, 2]. Therefore, detailed knowledge of the kinase signaling process is essential for the understanding of diseases and the development of new therapies.

While kinase signaling has been studied for over 100 years using a variety of experimental methods, the recent generation of mass spectrometry-based phosphoproteomic profiling allows for an unprecedented global exploration of phosphorylation. Phosphoproteomics data analysis involves two major steps. The first step includes the identification, phosphosite localization, and quantification of phosphopeptides. The second step aims to translate phosphopeptide identification and quantification results into novel biological and clinical insights. Although analyses in the first step are typically performed by the proteomics cores using standardized computational tools, those in the second step require and can benefit from active involvement of biologists and clinicians.

A vast array of resources and tools are available to facilitate the interpretation of phosphopeptide identification and quantification results. However, each of these tools exists as a silo without connection to tools with complementary functions. In addition, many tools have overlapping functions but differ in underlying knowledge bases, algorithms, input and output format of data, accessibility, advantages, limitations, and maintenance. Although newly developed tools are usually compared to similar, previously published tools, comparisons often do not include real-world, biological use-cases. For example, inference of kinase activity based on the observed phosphorylation of its substrates is a powerful application of phosphoproteomics profiling, and multiple methods have been developed to address this need [3, 4]. However, there has been little validation of the methods and only one benchmarking study comparing a few of the methods has been published [3].

Biological and clinical scientists are in the best position to extract biologically and clinically relevant findings from phosphoproteomics data, however, they are rarely consulted for tool design input or requested to test the final product. Furthermore, there is no comprehensive list of tools to aid those using phosphoproteomic data in their research. Therefore, this article aims to provide a comprehensive collection of resources that can be used to gain insights from phosphoproteomic data, including knowledge bases of kinases and phosphatases, phosphorylation sites, kinase inhibitors, and sequence variants affecting kinase function, and bioinformatics tools that can predict phosphorylation sites in addition to the kinase that phosphorylates them, infer kinase activity, and predict the effect of mutations on kinase signaling. We perform some benchmarking comparisons to determine the best tool available and assess usability of the tools from the standpoint of typical biologists or clinicians.

## Main text

### Collection of knowledge bases and tools

The OMICtools resource (https://omictools.com) is a manually curated collection of bioinformatics tools [5]. This site was searched in July 2019 for tools using the words ‘kinase’, ‘phosphorylation’, ‘phospho’, or ‘phosphatase’. In addition, several more tools were collected from the literature. Only tools that were freely available, still accessible, and non-obsolete were included, and tools specific for organisms other than human were discarded. The year of last update was assumed to be the year of publication unless otherwise noted on the website. These tools may be accessed by a downloadable, locally-run tool (Tool) or by a website (Web) that may have downloadable (DL) results or database information. The website URLs for all resources can be found in Additional file 1: Table S1. Each website was accessed in July 2019 and data statistics were collected for human proteins from downloadable files where possible and from websites or manuscripts for online-only resources.

### Knowledge bases of kinases and phosphatases

General information about the components involved in kinase signaling is required throughout the analysis and interpretation of phosphoproteomics data. Knowledge bases for kinase signaling can be separated into those collecting information on the enzymes, and those collecting experimentally validated phosphorylation sites. Of the 16 different resources that collect information specifically on protein kinases and phosphatases, 13 provide data on kinases, while 5 provide data on phosphatases (Table 1). Only two resources, the Eukaryotic Protein Kinase & Protein Phosphatase Database (EKPD) and its updated version iEKPD contain information on both types of enzymes [6]. Most databases are only available as online websites, but some provide an option for downloading data (Table 1).

**Table 1:** Knowledge bases of human kinases and phosphatases

Name	Last update	Method of access	Version	Enzyme	Human enzyme number	References
KinWeb	2005	Web		Protein Kinases	519	[11]
Kinomer	2008	Web|DL	1	Protein Kinases	505	[8]
MOKCa	2008	Web		Protein Kinases	423	[14]
HuPho	2012	Web|DL		Phosphatases	313	[18]
KIDFamMap	2012	Web		Protein Kinases	399	[17]
EKPD	2013	Web	1.1	Protein Kinases and Phosphatases	676	[6]
KinBase	2014	Web		Protein Kinases	538	[7]
KinMutBase	2015	Web|DL	4	Protein Kinases	31	[10]
DEPOD	2016	Web|DL	1.1	Phosphatases	239	[19]
KinaseNET	2017	Web		Protein Kinases	>530	
Kin-Driver	2017	Web|DL	82	Protein Kinases	518	[15]
Phosphatome	2017	Web	3	Phosphatases	189	[20]
iEKPD	2018	Web|DL	2.0	Protein kinases and Phosphatases	695	[105]
KLIFS	2018	Web|DL	2.4	Protein Kinases	292	[16, 106]
KinG	2019	Web|DL		Protein Kinases	1502	[9]
ProKinO	2019	Web	2	Protein Kinases	538	[12]

The kinase knowledge bases can be further separated into two different types: those that include comprehensive data on all known protein kinases, and those that were developed for a specific purpose, such as collecting driver mutations in kinases (Kin-Driver). Notably, no kinase resource collects data on non-protein kinases. KinBase, which was developed by Gerard Manning, contains 538 protein kinases and is considered the primary source of human protein kinases and their classification [7]. Many other resources base their kinase list on KinBase.

Kinomer and KinG are general kinase sequence databases that provide very little other information [8, 9]. KinMutBase, a collection of disease-causing mutations in protein kinase domains, is outdated, contains data on only 31 kinases, and primarily consists of broken links [10]. KinWeb and EKPD provide gene and protein identifiers, classification, description, and sequence information, but these data can also be found in other resources. However, KinWeb does have prediction of the disulfide bonding state of cysteines in the protein, as well as prediction of alpha helices, and EKPD presents data in an easy-to-read format [6, 11].

Use of the remaining general resources depends on which data one wants to access. KinaseNET, ProKinO, and iEKPD contain the most comprehensive data on protein kinases, but KinaseNET and ProKinO are only available as online resources [12, 13]. They include protein sequences, links to the kinases in other databases (e.g., UniProt, Ensembl, Entrez), information on the kinase domains, expression in tissue, and disease associations. ProKinO specifically contains pathway information, mutations and their disease associations, chromosomal location of the kinase, and links to published manuscripts. KinaseNET includes PTMs, known binding partners, inhibitors, upstream kinases, downstream substrates, and information about regulation. KinaseNET provides all data on a single page, ProKinO requires more than 10 clicks on separate tabs and pages to obtain all information on a kinase, and iEKPD contains links for 13 additional annotations.

For disease studies, MOKCa and Kin-Driver specifically have data on protein kinase mutations [14, 15]. MOKCa has tissue specificity of mutations while Kin-Driver focuses on driver mutations and reports whether the mutation is activating or inactivating. KLIFS provides structural information for approximately half of the protein kinases bound to various ligands [16]. Finally, KIDFamMap combines structural data with known kinase inhibitors and diseases [17].

Because phosphatases are less well studied than kinases, there are fewer resources dedicated to their collection. EKPD and iEKPD provide the same information for phosphatases as they do for kinases. HuPho, however, was the first comprehensive collection of phosphatases and the database includes pathway and substrate data, as well as siRNA phenotype data and links to orthologs in other species [18]. DEPOD also includes pathways, substrates, and links to orthologs in addition to interacting partners and upstream kinases [19]. Finally, Phosphatome.Net is the phosphatase version of KinBase [20]. The website contains basic classification and sequence information.

### Knowledge bases of phosphorylation sites

Besides information about specific kinases and phosphatases, data on phosphorylation sites are important for studying the signaling process. Phosphorylation site databases collect information on the location of phosphorylated residues in proteins from experimental data. These experiments can be low-throughput or high-throughput. High-throughput phosphorylation site identifications are assigned by probability unlike the more stringent experimental validation in low-throughput experiments, but some databases combine sites from both types of experiments without identifying the source experiment type.

In addition to phosphorylation site information, 16 of the 27 (60%) resources collect interactions between kinases or phosphatases and their substrates (Table 2). These often do not include the exact phosphorylation site, but instead provide interactions between an enzyme and its substrate at the gene level.

**Table 2:** Databases of phosphorylation sites

Name	Last update	Method of access	Version	Sites	Proteins	Kinases	Phosphatases	Data Type	References
PhosphoPep	2007	Web|DL	2.0	3980				MS	[30, 31]
HPRD	2010	Web|DL	9	78,005	11,807	291	42	UNSP	[21, 107, 108]
Phospho.ELM	2010	Web|DL	9.0	26,651	5374	250		HT, LT	[24, 109, 110]
Phospho3D	2010	Web|DL	2.0	1770		59		HT, LT	[44]
PHOSIDA	2011	Web|DL	3.24	24,262	8283			MS	[26, 111]
HuPho	2012	Web|DL		190	121		55	UNSP	[18]
PTMfunc	2012	Web		31,165				MS	[32]
ANIA	2013	Web|DL		305	220			LT	[35, 112]
PhosphoNetworks	2013	Web|DL			1140	255		UNSP	[36]
RegPhos	2013	Web|DL	2.0	66,301	10,849	380		UNSP	[42, 113]
Kinome NetworkX	2014	DL		173,460	18,610	357		UNSP	[41]
ProteomeScout	2014	Web|DL	2	290,007	23,387			MS	[39, 114]
LymPHOS	2015	Web|DL	2	15,566	4937			MS	[27, 115]
PhosphoDB	2015	Web		25,864	6222			MS	[29]
dbPAF	2016	Web|DL	1.0	244,034	18,773			UNSP	[38]
DEPOD	2016	Web|DL	1.1	253	210		88	UNSP	[19]
KANPHOS	2016	Web	β			73		MS	[25]
PhosphoAtlas	2016	DL		2595	1284	501		UNSP	[1]
Phosphopedia	2016	Web	1.0	109,611	11,428			MS	[28]
Phosphatome	2017	Web	3	6008	2000	319	106	UNSP	[20]
PhosphoNET	2017	Web		966,817^a^	22,698	488		UNSP + pred	[43]
PTMD	2018	Web|DL	1.0	690	434			UNSP	[37]
qPhos	2018	Web|DL		199,071	18,402			MS	[33]
dbPTM	2019	Web|DL	v2019	257,527	19,713	25		UNSP	[40, 64, 116, 117]
PhosphoSitePlus	2019	Web|DL	Aug-19	239,664	20,115	372		HT, LT	[23]
Signor	2019	Web|DL	May-19	3593	1285	336	70	UNSP	[34, 118]
Swiss-Prot	2019	Web|DL	Jun-19	40,135	7965	352		UNSP	[22, 119]

The number of unique kinases and phosphatases reported to phosphorylate sites in the database is included. For some databases, these numbers include enzyme groups in addition to individual enzymes. Data type indicates whether the data are from mass spectrometry (MS) experiments, separated high-throughput (HT) and low-throughput (LT) experiments, or whether the database combines data from both HT and LT experiments without specifying (UNSP)

^a^Indicates inclusion of predicted phosphorylation sites (pred)

**Fig. 1:**
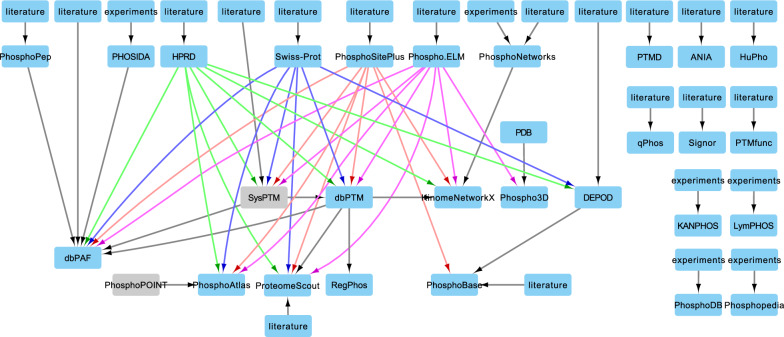
Network of phosphorylation site and kinase-substrate interaction databases. Gray nodes indicate databases that are no longer accessible. Arrows point from the knowledge source to the collecting database. Arrows originating from the four most highly used databases are colored by source (green = HPRD, blue = Swiss-Prot, red = PhosphoSitePlus, pink = Phospho.ELM)

The four main resources for phosphorylation sites curated data manually from the literature (Fig. 1). HPRD and Swiss-Prot are general databases of all proteins [21, 22]. The remaining two, PhosphoSitePlus and Phospho.ELM, specifically contain phosphorylation site information [23, 24]. Both PhosphoSitePlus and Swiss-Prot are frequently updated, while HPRD and Phospho.ELM were last updated in 2010. All four of these databases also include kinase information for sites if known.

Other smaller databases were generated through manual curation or publication of a laboratory’s own phosphorylation site data. KANPHOS collects phosphorylation sites in neural signaling identified by high-throughput experiments [25]. LymPHOS, PhosphoDB, Phosphopedia, and PHOSIDA are collections of data that were primarily produced in cell lines [26–29]. PhosphoPEP integrates mass spectrometry experiments from Cell Signaling Technology and their own laboratory [30, 31]. PTMfunc and qPhos both collect mass spectrometry experiments and add functional predictions and kinase activity from various tools [32, 33]. Signor extracts high quality signaling interactions from the literature [34]. Finally, ANIA, PTMD, and PhosphoNetworks curate the literature for a specific purpose. ANIA collects phosphorylation sites that serve as binding sites for 14-3-3 proteins, while PhosphoNetworks creates a kinase-substrate network curated from the literature and a protein microarray experiment, and PTMD collects disease-related phosphorylation sites [35–37].

The remaining resources integrate phosphorylation sites and kinase information from other databases (Fig. 1). The database dbPAF collects phosphorylation sites from several databases [38]. ProteomeScout also collects phosphorylation sites from other databases along with literature-curated experiments and provides a tool for analyzing a user’s data [39]. The database dbPTM collects all PTMs and the responsible enzyme from several sources [40]. Kinome NetworkX, RegPhos, and PhosphoAtlas curate and integrate data specifically to create kinase-substrate networks [1, 41, 42]. PhosphoNET is an online-only tool that includes predicted phosphorylation sites in addition to those with experimental evidence [43]. Finally, Phospho3D specifically collects phosphorylation sites with 3D structures [44].

Five databases collect information on phosphatase-substrate interactions. As mentioned, DEPOD, HuPho, and Phosphatome.Net all curate enzyme interactions from the literature. HPRD and Signor also collect some site-specific phosphatase information.

Each database contains a different number of phosphorylation sites and enzyme–substrate relationships depending on the source and method of collection (Table 2). ProteomeScout, PhosphoSitePlus, dbPTM, and dbPAF contain the most experimentally validated, downloadable sites. The site numbers for these four databases include specific protein isoforms, as do several other resources. PhosphoAtlas contains substrates for the largest number of individual kinases. Signor, Swiss-Prot, RegPhos, Phospho3D, dbPTM, and Phospho.ELM have substrates for individual kinases and kinase families. Finally, PhosphoSitePlus has substrates for some specific kinase isoforms.

### Errors in substrate databases

Based on our examination, PhosphoSitePlus is the preferred resource for experimentally-identified phosphorylation sites and kinases for phosphorylation sites. PhosphoSitePlus is frequently updated, well-curated, and distinguishes between low and high-throughput identified sites. The downstream integrating databases suffer from ID mapping errors. For example, in PhosphoAtlas there is an entry for PEG (paternally expressed gene 3) phosphorylating CDC25B. PEG is not a known kinase, but pEg3 kinase (also known as maternal embryonic leucine zipper kinase, *MELK*) is known to phosphorylate CDC25B [45]. Many of the downstream databases also have issues with PDPK1 and PDK1. The gene *PDPK1*, 3-phosphoinositide-dependent protein kinase 1, produces a protein known to the biological community as PDK1. However, there is an additional kinase, pyruvate dehydrogenase kinase, that is produced by the gene *PDK1*. Databases that try to integrate sites frequently attribute the substrates of *PDPK1* to *PDK1*. Finally, integrating databases propagate errors from the original databases. For example, HPRD contains an entry for PTPN11 phosphorylating PTK2B although PTPN11 is a known phosphatase and not a kinase. The original manuscript connected to this entry confirmed that PTPN11 is a phosphatase and that it just binds to PTK2B at that particular site [46]. Databases that collect information from HPRD, such as RegPhos and PhosphoAtlas, include this incorrect entry for PTPN11.

### Known substrates of kinases and phosphatases

**Fig. 2:**
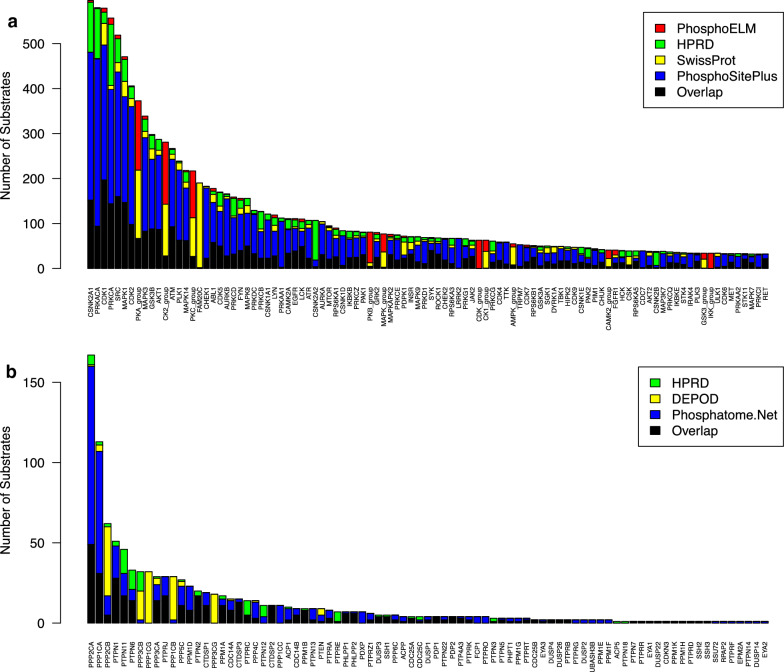
Number of substrates per kinase and phosphatase. **a** Number of substrates for the top 100 kinases in four databases. Substrates present in more than one database are colored black while the remaining sites are unique to each database. **b** Number of substrates for each phosphatase in DEPOD (yellow), HPRD (green), Phosphatome.Net (blue), or in more than one database (black)

The four main databases of kinases together produce 485 substrate sets of individual kinases and kinase families (Fig. 2a). PhosphoSitePlus contains the most unique sites, while other databases contribute only a few additional sites per kinase. CSNK2A1 has the most substrates (596), while over half of the sets contain fewer than 10 substrates.

For substrates of phosphatases, DEPOD, HPRD, and Phosphatome.Net combined produce sets for 83 phosphatases. The most unique information comes from DEPOD and Phosphatome.Net. The number of known sites for each phosphatase is far fewer than that for kinases. PPP2CA has the most substrates (167), while 70% of the phosphatases have fewer than 10 substrates (Fig. 2b).

### Phosphorylation site prediction tools

Despite decades of research, very few phosphorylation sites have known kinases or phosphatases. Of the sites in PhosphoSitePlus, only about 3% have an experimentally validated human kinase. Therefore, numerous tools have been developed to predict which sites in a protein can be phosphorylated and which kinases phosphorylate that given site.

These prediction tools were developed using a variety of features and methods and have been reviewed elsewhere [47, 48]. The early versions of phosphorylation site predictors were motif-based. They generated the frequency of amino acids surrounding a site and searched for that pattern in protein sequences. Later tools used more sophisticated methods such as support vector machines (SVM), random forest, Bayesian probability, position specific scoring matrices (PSSM), and deep neural networks [49–53]. Besides amino acid sequence, tools included a vast array of features such as the 3D structure of the phosphorylation site, disorder score, cell cycle data, and co-expression of kinases and substrates [54–56]. Others, like NetworKIN and iGPS, used protein–protein interaction data to filter predictions [57, 58]. Table 3 provides an overview of all currently available tools to predict phosphorylation sites or kinases for phosphorylation sites. While a few tools have been developed to predict sites for phosphatases, only Ptpset, NetPhorest, and NetworKIN are still accessible [49, 58].

**Table 3:** Available phosphorylation site and kinase-substrate prediction tools

Tool	Last update	Version	Prediction type	Method	Kinases/phosphatases	Type	References
DISPHOS	2004	1.3	Phosphorylation sites	Bagged logistic regression	0	Web	[54]
PPSP	2006	1.06	Phosphorylation sites of kinases	Bayesian decision theory	68	Web	[52]
KinasePhos2.0	2007	2.0	Phosphorylation sites of kinases	SVM	58	Web	[51]
pkaPS	2007		Phosphorylation sites of PKA	Scoring function	1	Web|DL	[72]
PhoScan	2008		Phosphorylation sites of kinases	Scoring function	48	Web|Tool	[60]
Phos3D	2009		Phosphorylation sites and some kinase specificity	SVM	5	Web	[55]
Musite	2010	1	Phosphorylation sites and some kinase specificity	SVM	13	Web|DL	[62]
PHOSIDA Predictor	2011	3.24	Phosphorylation S and T sites	SVM	0	Web	[26]
Predikin	2011		Phosphorylation sites of kinases	PSSM	any	Web|DL	[53]
GPS-Polo	2012	1.0	Phosphorylation sites of Plk	Group-based scoring function PSSM	1	Web|Tool	[120]
iGPS	2012	1.0.1	Phosphorylation sites of kinases in vivo	GPS with PPI	407	Tool	[57]
CEASAR	2013		Kinases for known phosphorylation sites	Naïve Bayes	289	DL	[56]
HMMpTM	2013		Phosphorylation sites of kinases and topology	HMM	9	Web|DL	[121]
PKIS	2013		Phosphorylation sites of kinases	SVM	56	Web	[122]
GPS	2014	5.0	Phosphorylation sites of kinases	Group-based scoring function PSSM	464	Web|DL|Tool	[61]
NetPhorest	2014	2.1	Phosphorylation sites of kinases	ANN and PSSM	244	Web|DL|Tool	[58, 65]
NetworKIN	2014	3.0	Phosphorylation sites of kinases in vivo	Naïve Bayes with PPI	123	Web|DL|Tool	[58, 66]
phos_pred	2014		Predicts phosphorylation sites for kinases	Random forest	54	Tool^a^	[49]
PhosphoSVM	2014		Phosphorylation sites	SVM	0	Web	[123]
Ptpset	2014		Dephosphorylation sites of phosphatases	KNN	3	Web	[124]
jEcho	2015	1.0	Phosphorylation sites of kinases	Weight vector	12	Tool	[68]
KSP-PUEL	2015		Phosphorylation sites of kinases	SVM ensemble	2^a^	Tool	[63]
Scansite	2015	4	Kinase motifs in proteins	PSSM	70	Web|DL	[125]
DAPPLE	2016	2	Phosphorylation sites	BLAST	0	Web|DL	[126]
iPhos-PseEn	2016		Phosphorylation sites	Random forest ensemble	0	Web	[127]
PhosphoPICK	2016		Phosphorylation sites of kinases	Bayesian network	107	Web|DL	[70]
PhosD	2016		Kinase-substrate relationships	Probabilistic model	399	DL	[128]
MusiteDeep	2017		Phosphorylation sites and some kinase specificity	DNN	5	Tool^a^	[50]
NetPhos	2017	3.1	Phosphorylation sites and some kinase specificity	ANN	17	Web|Tool^a^	[59, 71]
PhosphoNET	2017		Phosphorylation sites of kinases	PSSM	488	Web	[43]
PhosPred-RF	2017		Phosphorylation sites	Random forest	0	Web	[129]
AKID	2018		Phosphorylation sites of kinases	DNN	496	Web|DL|Tool^a^	[69]
CapsNet_PTM	2018		Phosphorylation sites and some kinase specificity	DNN	any	Tool	[130]
PTM-ssMP	2018		Phosphorylation sites and some kinase specificity	SVM	9	Web|DL	[131]
Quokka	2018		Phosphorylation sites of kinase families	Logistic regression	65	Web|DL	[132]
TyrPred	2018		Phosphorylation sites of Tyr kinases	Elastic net	15	Web	[133]
CoPhosK	2019		Kinases of phosphorylation sites	Naïve Bayes	101	Web|Tool	[134]
DeepPhos	2019		Phosphorylation sites and some kinase specificity	DNN	20	Tool	[67]

^a^Indicates number of trained kinases, but tool can be trained with others

*SVM* support vector machine, *PSSM* position specific scoring matrix, *GSEA* gene set enrichment analysis, *ANN* artificial neural network, *DNN* deep neural network, *HMM* hidden Markov model, *PPI* protein–protein interaction, *KNN* K-nearest neighbor

^a^Indicates tool is not available for all three main operating systems (Linux, Mac, Windows)

**Fig. 3:**
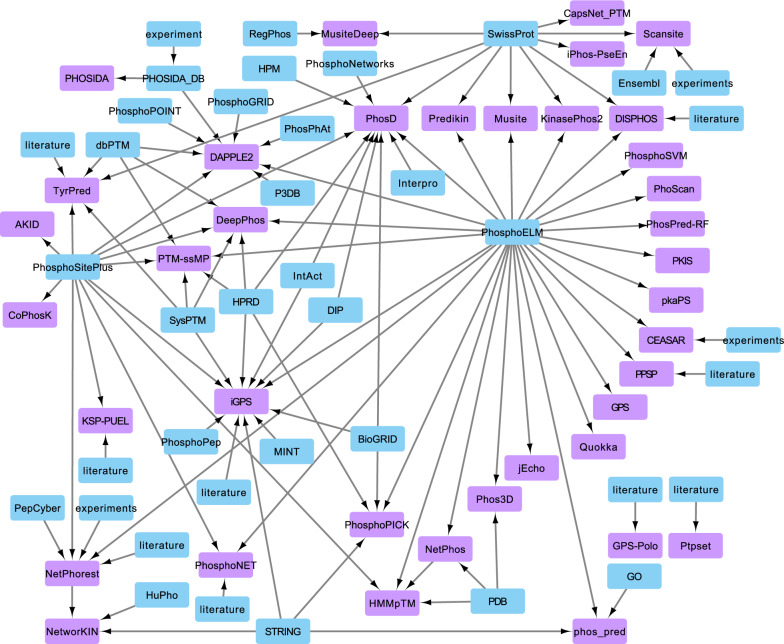
Network of phosphorylation site predictor tools and the resources used to make predictions. Tools are colored purple while the databases used by the tools are colored blue

Figure 3 shows phosphorylation site predictor tools and the resources they used to make predictions. Almost all phosphorylation site predictors were trained using data from Phospho.ELM. Swiss-Prot and PhosphoSitePlus were also heavily used resources. Notably, almost all tools were developed using experimentally verified substrate data as the training set. Therefore, the tools are only able to predict the responsible kinase if there is sufficient data for substrates of that kinase.

A researcher may utilize these prediction tools to identify kinases phosphorylating single substrates of interest, for which web-based tools would suffice. However, the limit on the number of sequences submitted for prediction and the lack of downloadable results prevent these same tools for being useful in large-scale phosphoproteomic studies. Unfortunately, many tools appropriate for large-scale studies have multiple issues limiting their use. First, tools can be difficult to install, platform-specific, and lack manuals on use. For example, NetPhos [59] is downloadable but can only be run on Linux, whereas PhoScan [60] can only be run on Windows machines. Other tools require commercial software such as MATLAB or even require understanding a programming language to modify hard-coded variables. Finally, tools like GPS [61] and phos_pred [49] provide pre-defined cutoffs for prediction, while others like musite [62] and KSP-PUEL [63] allow users to define their own thresholds or to train the models using their own data.

### Testing kinase-substrate relationship prediction tools

For large-scale kinase-substrate prediction, 14 pre-trained tools were available that provide downloadable results. The best, unbiased way to test these tools is to use validated sites that were not used for the training of any tool. Unfortunately, most tools do not report the actual sites used for training and finding a set of sites to fit these criteria is nearly impossible. Therefore, we evaluated all 14 tools using gold-standard positive and negative human phosphorylation sites downloaded from dbPTM [64] for four serine/threonine kinases (CDK1, CK2, MAPK1, and PKA). Positive sites were serines and threonines experimentally validated to be phosphorylated by a particular kinase. Negative sites were serines and threonines not known to be phosphorylated on the same proteins. The outcomes might be biased in favor of newer tools and those that used some of these sites in their training.

Tools predicting kinases for phosphorylation sites (Table 3) were accessed through local tool installation or through the tool’s website. PhoScan [60] and phos_pred [49] were run locally on a Windows laptop, while NetPhorest [65], NetworKIN [66], iGPS [57], GPS [61], DeepPhos [67], jEcho [68], and MusiteDeep [50] were run locally on a Mac laptop. AKID [69], PhosphoPICK [70], NetPhos [71], Musite [62], and pkaPS [72] were accessed via their websites. Tools were set with the lowest threshold if they did not have an option to return scores for all sites. For each site, the maximum score was retained if the tool predicted for more than kinase isoform (e.g., the maximum score of PKCalpha and PKCbeta on the same site). If a tool did not return a score for a site, the lowest possible score was given to the site. The receiver operating characteristic (ROC) curve and area under the ROC curve (AUROC) were calculated for the results from each tool using the R package ROCR [73].

**Fig. 4:**
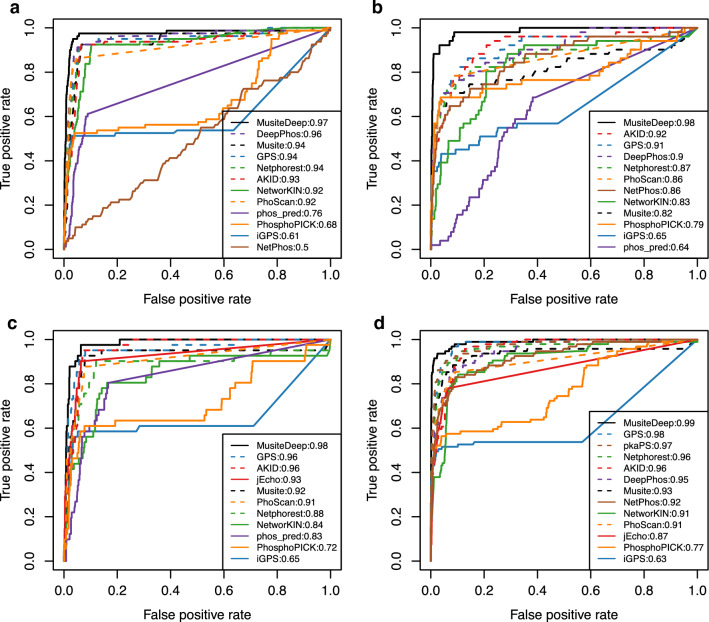
ROC curves for substrate prediction of four kinases. The false positive and true positive rates of substrate prediction for **a** CDK1, **b** CK2, **c** MAPK1, and **d** PKA. The AUC for each tool is listed next to the tool name

ROC curves for four kinases (CDK1, CK2, MAPK1, and PKA) are shown in Fig. 4. Notably, musite was unable to predict for a few random protein sequences in each submission. DeepPhos and phos_pred both required manual edits of hard-coded variables. MusiteDeep and GPS had the highest area under the curve (AUC) for all kinases tested. The PKA-specific tool pkaPS also performed well. Performance for most tools, however, varied across kinases.

### Comparison of kinase activity tools

The known or predicted kinases for phosphorylation sites can be used to infer kinase activity from global phosphoproteomic data. Tools and methods have been developed to predict kinase activity, but there has been little effort spent towards comparing these tools or determining the most biologically-relevant set of parameters. The available tools (PHOSIDA, KEA2, KSEA App, PHOXTRACK, INKA, and IKAP) each use a different algorithm to infer activity (Table 4). The PHOSIDA de novo motif finder uses a simple method of bootstrapping to determine enrichment of sequence motifs in a set of phosphorylated peptides and then matches those to known kinase motifs [26]. Kinase Enrichment Analysis 2 (KEA2) uses over-representation analysis to determine enrichment of kinase substrates in a condition [74]. Similarly, the KSEA App uses mean phosphorylation of substrates of kinases as a proxy for activity [4]. PHOXTRACK modified pre-ranked gene set enrichment analysis (GSEA) to determine enrichment of known kinase targets [75]. IKAP extended these methods using a cost function to infer the relative contributions of multiple kinases acting on the same site [76]. Finally, INKA combines the GSEA method with activating phosphorylation on kinases [77].

**Table 4:** Kinase activity prediction and phosphoproteomic dataset analysis tools

Tool	Last update	Prediction type	Method	Input	Type	References
PHOSIDA Motif Finder	2011	Sequence motifs	Bootstrap	Phosphosite 13mer	Web	[26]
CellNOpt	2012	Time-course analysis	Logic formalisms	Interactions and phosphoproteomic data	Tool	[85]
KEA2	2012	Kinase activity	Fisher’s exact test	Gene symbols and phosphosite	Web|DL|Tool	[74]
Sorad	2013	Time-course analysis	Ordinary differential equations	Phosphoproteomic data	Tool	[86]
PHOXTRACK	2014	Kinase activity	GSEA	Phosphosite 13mer and log2 expression	Web|DL	[75]
PhosFox	2015	Phosphorylation site comparison between groups	Comparison	Phosphoproteomic data	Tool	[84]
SELPHI	2015	Phosphoproteomic data analysis	Multiple functions	Phosphoproteomic data	Web|DL	[83]
DynaPho	2016	Phosphoproteomic analysis for multiple conditions	Activity modules	phosphoproteomic data	Web|DL	[87]
IKAP	2016	Kinase activity	Cost function	Phosphoproteomic data	Tool	[76]
KinasePA	2016	Kinase perturbation in multiple treatments	Directional hypothesis testing framework	Phosphoproteomic data	Web|Tool	[88]
KSEA	2017	Kinase activity	Z score	Phosphoproteomic data	Web|DL|Tool	[4]
CLUE	2018	Time-course kinase activity	k-Means clustering	Phosphoproteomic data	Tool	[41]
INKA	2019	Kinase activity	GSEA and phosphorylation	Phosphoproteomic data	Web|DL	[77]

*GSEA* gene set enrichment analysis

We used a phosphoproteomic dataset from a cell line experiment with 20 kinase inhibitors [78] to test four kinase activity prediction tools. Because PHOSIDA is only available online without downloadable results, we excluded this tool from further analysis. INKA was also excluded as it requires MaxQuant search result files. The R programming environment was used to create files in the input format for each tool. Significantly downregulated sites for each inhibitor were submitted to KEA2 and significantly inhibited kinases were defined as those with false discovery rate (FDR) < 0.05 and at least 3 overlapping substrates [74]. The log2 fold change for each thirteenmer phosphorylation site (± 6 amino acids surrounding the phosphorylated site) was submitted to PHOXTRACK (1000 permutations, minimum number of substrates = 3, weighted statistics) [75]. Significantly inhibited kinases were defined as those with FDR < 0.05 and normalized enrichment value < 0. The fold change for each site with each inhibitor was submitted to the KSEA app website and significantly inhibited kinases were defined as those with FDR < 0.05, at least 3 substrates in the dataset, and a z score < 0 [4]. The substrates of kinases from PhosphoSitePlus (version July 2017) and Signor (version October 2017) were used for IKAP [23, 34, 76]. IKAP was run locally on a Mac laptop with the bounds between -11 and 11 and 50 iterations. The 5 kinases with the lowest activity scores for each experiment were chosen. The positive set were kinases known to be inhibited by each drug (as reported in supplementary table in Ref. [78]); all other kinases predicted by the tools were considered to be negative. The significant kinases for each tool were counted for presence in the positive and negative sets.

Comparison of these tools is challenging because they use different input and underlying databases. KEA2 requires a set of sites in the format of HGNC symbol and phosphorylated amino acid residue position separated by an underscore. It contains sets for 250 different kinases. KSEA App requires a strictly formatted comma-delimited file with the HGNC symbol, phosphorylated position, and non-log-transformed fold change. Users can choose between known sets from the July 2016 release of PhosphoSitePlus or the known + predicted site sets from PhosphoSitePlus and NetworKIN. PHOXTRACK requires a two-column file with a thirteenmer peptide and log-transformed fold change. It can use substrate sets from the four main databases or a user-supplied database. Finally, IKAP required tabular data entered into MATLAB, manual modification of MATLAB code to change parameters, and allowed a user to upload their own set of substrates. Because one thirteenmer might match multiple proteins and phosphorylated positions, the actual substrate list presented to each tool may differ slightly.

**Fig. 5:**
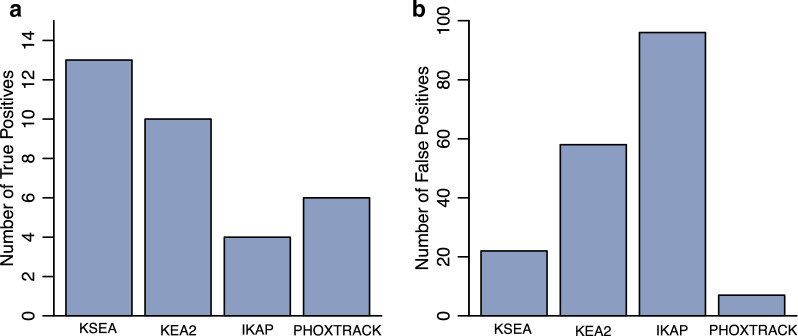
True and false positive predictions for kinase activity prediction tools. **a** For all 20 inhibitors, the number of known targets predicted to be significantly downregulated by each tool. **b** For all inhibitors, the number of all significantly downregulated kinases that do not match known inhibitor targets

To determine how well each tool covered the known targets of kinases, we counted the number of significantly downregulated known kinases of each inhibitor and the significantly downregulated kinases of each inhibitor that were not known targets of that inhibitor. The KSEA App made the most true positive predictions across all experiments, while IKAP made the fewest true positive predictions (Fig. 5a). PHOXTRACK made the fewest false positive predictions (Fig. 5b).

Besides upstream kinase activity, phosphoproteomics data could additionally be used to explore altered downstream pathways. While standard tools and methods such as GSEA are typically used for this analysis, all are limited to using overall gene-level phosphorylation [79]. Unfortunately the functional contribution of individual sites to pathway signaling is poorly annotated in gene set databases, although PTMsigDB has some limited pathway sets [80]. Until new tools are built to handle individual sites in pathway analysis, a user might combine the results from kinase activity prediction to assemble altered kinases into pathways using tools such as String, RegPhos2, or Wikipathways [42, 81, 82].

### Differential and clustering analysis of phosphoproteomics data

Besides activity prediction, phosphoproteomic data can be used for other analyses. SELPHI is a good tool to first explore the data as it allows biologists to quickly and easily analyze phosphoproteomic data with clustering analyses, kinase-substrate correlation, and pathway enrichment [83]. PhosFox then compares phosphorylated peptides between conditions [84]. Finally, a set of tools (CellNOpt, Sorad, CLUE, DynaPho, and KinasePA) were developed specifically for phosphoproteomic time-course or multiple condition analyses (Table 4) [41, 85–88].

### Prediction of mutation effect

Analysis and interpretation of phosphoproteomic data can be enhanced with other multi-omics data types. For example, sequence variants can affect kinase function or presence of a phosphorylation site. The databases PhosSNP [89] and ActiveDriverDB [90] collect gene polymorphisms and somatic mutations, respectively, near phosphorylation sites and categorize them based on suspected effect (Table 5). ActiveDriverDB also includes predictions from Mutations Impact on Phosphorylation (MIMP), which uses Bayesian statistics to predict whether mutations around a phosphorylation site will change which kinase binds to that site [91]. It can predict rewiring for 124 kinases using experimentally validated data, or it can be extended to predict for 322 kinases using predicted kinase-substrate relationships. ReKINect also predicts rewiring from mutations, but it further predicts the destruction or creation of phosphorylation sites and inactivation or constitutive activation of kinases [92]. PhosphoPICK-SNP is also similar to MIMP. It predicts the kinase responsible for phosphorylating a site, and whether a mutation affects its ability to phosphorylate the site [93]. While all of the tools are easy to use, the databases are better for individual searches and the three prediction tools are better for analysis of a user’s mutation data.

**Table 5:** Resources for studying the effect of mutations on kinases and phosphorylation sites

Tool	Last update	Version	Prediction type	Method	Kinases	Method of access	References
PhosSNP	2009	1.0	SNVs that might influence phosphorylation status	Rules		Tool	[89]
MIMP	2015		Missense SNV impact on kinase-substrate	Bayesian model	322	Web|DL|Tool	[91]
ReKINect	2015		Effect of SNV on signaling network	PSSM		Web|DL	[92]
PhosphoPICK-SNP	2016		Effect of SNV on phosphorylation level	Bayesian models	107	Web|DL	[93]
ActiveDriverDB	2017		Somatic variants affecting phosphorylation	Rules	322	Web|DL	[90]

*SNV* single nucleotide variation, *PSSM* position specific scoring matrix

### Resources for kinase inhibitors

After discovering altered kinases from phosphoproteomic data to use as therapeutic targets, identifying inhibitors is essential. Most available resources connect known drugs to their known kinase targets (Table 6). DrugKiNET shows the known inhibitors for kinases, and the kinases that a compound inhibits. It also predicts which kinases a drug can inhibit. K-Map extends these interactions to suggest the best compound to inhibit a set of kinases [94]. Finally, KinomeSelector groups kinases by sequence similarity and similarity of drug response. It then allows a user to choose a subset of kinases to target that cover the kinome [58].

**Table 6:** Kinase-inhibitor relationship resources

Tool	Last update	Description	Kinases	Inhibitors	Method of access	References
K-Map	2013	Best inhibitor for a set of kinases	300 or 442	178 or 72	Web|DL	[94]
KinomeSelector	2014	Minimal set of kinases to inhibit	> 500	NA	Web|DL	[58]
DrugKiNET	2017	Known and predicted drug activity on kinases	> 800		Web|DL	

K-Map has two different databases—one with 178 drugs inhibiting 300 kinases and one with 72 drugs inhibiting 442 kinases

### Other kinase signaling tools

The final set of bioinformatics tools, summarized in Table 7, enhance phosphoproteomic analysis and cover visualization, data retrieval, and prediction tools. Additional kinases from a genome can be predicted by Kinannote [95] and KinConform can predict whether those kinases are active in structure files [96]. KinMap [97] is used to visualize the entire kinome tree and PhosphoLogo [98] is used to generate sequence logos of kinases. On the other side, RLIMS-P and eFIP are both tools that extract data on phosphorylation interactions from the literature [99, 100]. Then CPhos identifies phosphorylation sites of interest that are conserved across species [101]. PyTMs [102] is a tool to visualize 3D structures of phosphorylation sites and ultimately RegPhos2.0 [42] can be used to visualize signaling networks. RegPhos2.0 also provides heatmaps for kinase and substrate mRNA expression in cancer. Finally, 14-3-3-Pred predicts phosphorylation sites in protein sequences that might bind to 14-3-3 proteins, further adding to the phosphorylation-related signaling network [103].

**Table 7:** Visualization, data retrieval, and prediction tools

Tool	Last update	Version	Type	Input	Output	Method of access	References
CPhos	2012	1.3	Phosphorylation site conservation	Phosphopeptides	Conservation scores	Tool	[101]
PhosphoLogo	2012		Visualization of sequence motifs	Sequence motifs	Motif visualization	Tool	[98]
RegPhos2.0	2013	2.0	Visualization of kinase data	Gene names	Network visualization or cancer gene expression	Web|DL	[42, 113]
eFIP	2014		Returns publications involving phosphorylation	Gene names or words	Publications matching those words	Web	[100]
RLIMS-P	2014	2.0	returns protein phosphorylation information from literature	PMIDs or keywords	kinase, substrate, and site	Web|DL	[99]
PyTMs	2015	1.2	pyMOL plugin to add PTMs to protein models	Protein models, PTMs	PTMs integrated in protein models	Tool	[102]
14-3-3-Pred	2015		Predicts 14-3-3 binding phosphosite	Protein sequences	Predicted 14-3-3 binding sites	Web|DL	[103]
KinMap	2016		Kinome tree visualization	Kinases	Tree with highlighted branches	Web|DL	[97]
KinConform	2017		Determines which structures are kinases	Structures	Active or inactive kinase chains	Tool	[96]
Kinannote	2017	1.0	Classifies sequences as kinases	Protein sequences	Kinase annotation	Tool	[95]

## Discussion

The available databases and tools for studying kinase signaling cover diverse functions and include information on enzymes and their substrates, inhibitors, activity, and mutations. Together these knowledge bases, prediction tools, and analysis tools comprise the current best standard for studying kinase signaling and many can be used without extensive computational knowledge. Overall, these tools allow a researcher to discover vast amounts of information from their phosphoproteomic data and some tools can even perform entire sets of analyses with a single button click [83].

Despite the work that has been done, there is room for advancement to fully utilize phosphoproteomic data for use in the clinic. First, the majority of tools focus almost exclusively on the study of protein kinases. However, phosphatases are critical components of the kinase signaling cascade and are frequently dysregulated in cancer. Understanding the role of the interplay between kinases and phosphatases on the net phosphorylation seen in global phosphoproteomic data is essential to identifying abnormal cell signaling in disease. Furthermore, while the current tools and research are aimed at studying dysregulated protein phosphorylation, non-protein phosphorylation is also often altered in disease. For example, hexokinases, which phosphorylate glucose, drive glucose metabolism and contribute to tumor initiation in mouse models of lung and breast cancers [104]. The development of resources and tools to study non-protein kinases and phosphatases could advance research in a variety of fields.

While the current tools provide critical functions, their error rate and accuracy could be improved. Errors are frequently propagated or amplified when tools collect data from a variety of resources. However, the impact of these errors on downstream analyses and biological inferences remains to be determined.

For all tools, usability can be an issue, both for bioinformaticians and biologists with no computational experience. Tools are frequently platform-dependent, do not allow downloadable results, and are not well annotated. Furthermore, tools are difficult to compare or to use more than one during analysis. The input and output formats are not standardized and use a variety of protein naming conventions.

The largest challenge was deciphering input limitations and understanding results. For example, submitting a sequence with a large number of phosphorylatable residues to GPS caused the software to stall without an error message and no documentation mentioned a size limit. Musite did not provide results for a sequence or two each run without explanation. Furthermore, downloadable result files for many tools had no column headers so the column contents were unknown. For example, the downloadable file from musite has no column titles, so you have to check the table on the website to understand the results. Additionally, scores are usually presented without explanation. Only careful reading of the manuscript or the manual elucidates what value signifies a “good” response. For example, in Scansite, the score 0 is the best, with scores closest to 0 indicating the best match. But in PhosphoPICK, the score indicates the probability of being phosphorylated by a kinase at that site so a score closer to 1 is better. Experts in machine learning might understand the score without explanation, but naïve users likely will not.

One way to fix this challenge is to have a detailed, easy-to-find manual. The manual should include ways to run the tool, the underlying mechanism of the method, and detailed description of the results. The description of the results should also be available where results are visualized. Furthermore, sample input is helpful for a new user to test the tool and determine whether the results will be useful for their experiment before preparing their own data files.

## Conclusions

There are many tools and resources that can be used to study kinase signaling and these tools will become even more essential with the continued production of phosphoproteomic data. It is essential for the biological community to research under-studied enzymes and to validate specific substrates of kinases and phosphatases. Furthermore, bioinformaticians should consider creating tools that utilize information from both sides of the enzymatic phosphorylation reaction. Finally, resources should be carefully planned, easy to use, and well maintained and the community should work to standardize the use of enzyme IDs and phosphorylation site location.

### Supplementary information

**Additional file 1.** List of URLs for all resources.

## Data Availability

The data used for comparing kinase activity inference tools can be found in PubMed with PMID: 28674151.

## Abbreviations

ANN: Artificial neural network
AUC: Area under the curve
AUROC: Area under the ROC curve
DL: Downloadable
FDR: False discovery rate
GSEA: Gene set enrichment analysis
HMM: Hidden Markov model
HT: High-throughput
LT: Low-throughput
MELK: Maternal embryonic leucine zipper kinase
PDK1: Pyruvate dehydrogenase kinase 1
PDPK1: 3-Phosphoinositide-dependent protein kinase 1
PEG: Paternally expressed gene 3
PPI: Protein-protein interaction
PSSM: Position specific scoring matrix
PTM: Post-translational modification
ROC: Receiver operating characteristic
SVM: Support vector machine
Web: Website
