# Correction to: Using phosphoproteomics data to understand cellular signaling: a comprehensive guide to bioinformatics resources

**DOI:** 10.1186/s12014-024-09473-w

**Published:** 2024-03-07

**Authors:** Sara R. Savage, Bing Zhang

**Affiliations:** 1 Department of Biomedical Informatics, Vanderbilt University, Nashville, TN USA; 2 Lester and Sue Smith Breast Center, Baylor College of Medicine, Houston, TX USA; 3 Department of Molecular and Human Genetics, Baylor College of Medicine, Houston, TX USA

Correction to: Clinical Proteomics (2023) 17:27

10.1186/s12014-020-09290-x

In the main text, under the section heading “Knowledge bases of kinases and phosphatases“, 6th paragraph, the 3rd sentence that reads as “DEPOD used data from HuPho as a starting point and therefore contains much of the same information [19]” should have read as “DEPOD also includes pathways, substrates, and links to orthologs in addition to interacting partners and upstream kinases [19]”. The original article has been corrected.
